# Herpes Zoster Ophthalmicus Complicated by Unilateral Ptosis and Abducens Nerve Palsy: A Case Report

**DOI:** 10.7759/cureus.25311

**Published:** 2022-05-25

**Authors:** Anna Mueller, Henrique Carvalho, Daniel Montenegro

**Affiliations:** 1 Ophthalmology, Florida International University, Herbert Wertheim College of Medicine, Miami, USA; 2 Ophthalmology, Eye Centers of South Florida, Miami, USA

**Keywords:** ophthalmoplegia, diplopia, ptosis, abducens nerve, herpes zoster ophthalmicus

## Abstract

Herpes zoster ophthalmicus (HZO) is a viral condition that presents as a painful vesicular rash in the trigeminal nerve dermatome. In some cases, self-limiting extraocular muscle palsies may occur several weeks after the onset of HZO and last for weeks to months. A 71-year-old man developed a debilitating binocular horizontal diplopia and ptosis about four weeks after the onset of HZO symptoms. He had no uveitis, keratitis, or changes in visual acuity. Examination revealed left abducens palsy and ptosis. Symptoms resolved within eight weeks without any intervention.

## Introduction

Herpes zoster ophthalmicus (HZO) is a neurocutaneous condition associated with the reactivation of varicella-zoster virus (VZV), also known as shingles, resulting in inflammation at the ophthalmic division (V1) of the trigeminal nerve. In the United States, around 20-30% of individuals develop shingles at some point in their lives [1]. HZO is responsible for 10-20% of all cases of shingles, with ophthalmoplegia presenting in 7-31% of HZO cases [2]. HZO incidence rate has increased in recent years, from 8.1 cases/100,000 persons in 2004 to 19.5/100,000 in 2016 [3]. Elderly and immunocompromised patients have a higher risk of developing HZO with more serious visual sequelae, which may require a longer course of treatment than younger, immunocompetent patients [4].

In the acute phase, HZO presents with constitutional symptoms of fatigue, fever, malaise, and a painful, unilateral, vesicular rash in the upper hemiface [5]. Ophthalmoplegia may occur weeks after the onset of HZO symptoms, classically presenting as diplopia. Diagnosis of HZO is clinical based on history and examination with a slit-lamp, ocular tonometry, or corneal esthesiometry. Treatment includes antiviral therapy. If left untreated, patients can develop severe complications, including permanent vision loss, necrotizing retinitis, and neurotrophic keratopathy [5]. Here, we present the case of a patient who developed binocular horizontal diplopia and ptosis four weeks after the onset of HZO symptoms.

## Case presentation

A 71-year-old Hispanic man without a significant medical history presented to our Miami clinic with a painful vesicular eruption on his left upper hemiface. He was diagnosed with HZO a week prior at the emergency department and was discharged with a two-week course of 1 g valacyclovir by mouth three times a day. A computerized tomography scan of the patient’s brain was performed at the emergency department, which was negative. Upon physical examination at our clinic, the patient’s visual acuity was 20/60 in the right eye and 20/30 in the left eye. His symptoms were consistent with HZO but displayed no evidence of keratitis or uveitis. The patient was instructed to complete his treatment of valacyclovir and return for a follow-up in two weeks unless he experienced visual changes or pain.

Two and a half weeks later, the patient returned with left-eye ptosis, periocular pain, and a watery left eye. He had already completed his two-week course of valacyclovir. On examination, the vesicular rash on his left upper hemiface had improved substantially. The patient was started on 500 mg of oral gabapentin twice a day for his postherpetic neuralgia.

**Figure 1:**
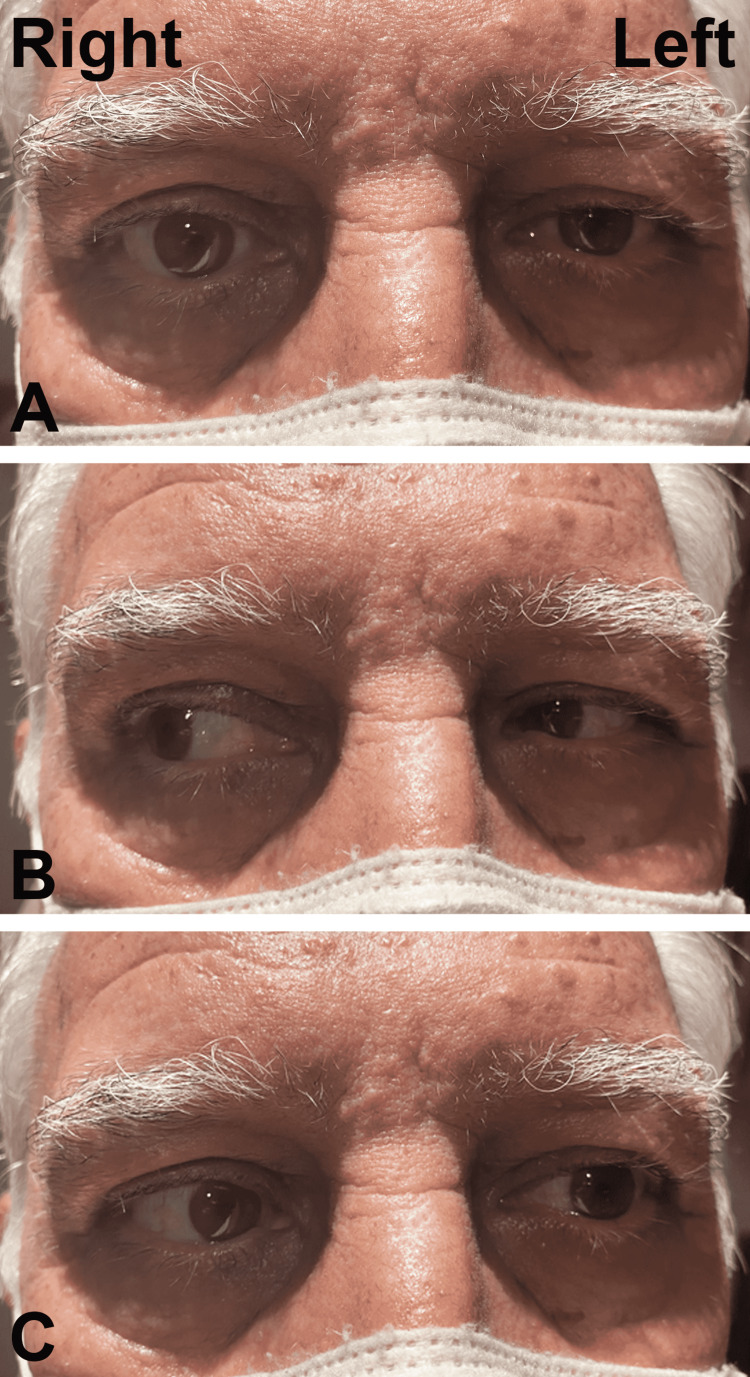
Four weeks after the onset of HZO symptoms. (A) Straight gaze with mild left ptosis. (B) Successful right gaze. (C) Limited abduction on left gaze. HZO: herpes zoster ophthalmicus

Two days later, the patient developed binocular horizontal diplopia, and further evaluation revealed left abducens nerve palsy (Figure 1). His left-eye ptosis persisted, but all other extraocular movements were intact. Pupils were equal, round, and responsive to light. No corneal involvement was identified. There were no changes in visual acuity. Fundoscopy was normal. There were no signs of HZO on the patient’s right eye. No additional treatment was prescribed at this time. 

In a follow-up three months after the onset of symptoms, the ptosis and abducens nerve palsy had completely resolved. The only remaining symptom was postherpetic neuralgia above the patient’s left eye.

## Discussion

HZO may lead to ophthalmoplegia weeks after the onset of the neurocutaneous symptoms. The most common cause of ophthalmoplegia appears to be oculomotor nerve palsy, which may present as diplopia, mydriasis, and ptosis [2,6]. Disruption to the cranial nerve’s supply of extraocular muscles leads to diplopia, while disruption to the levator palpebrae superioris muscle supply leads to ptosis. Lastly, mydriasis occurs when the parasympathetic fibers running along the nerve are affected. It is possible that our patient had some levator palpebrae superioris muscle weakness, as evident by the mild ptosis. There was no evidence of other oculomotor nerve dysfunction. Abducens nerve palsy is the second most common symptom of HZO-related ophthalmoplegia. It presents with impaired abduction of the ipsilateral eye [7], as was observed in our patient. Trochlear nerve palsy is the rarest cause of HZO-related ophthalmoplegia, impacting downgaze and causing vertical or oblique diplopia [8]. We did not observe any evidence of trochlear nerve involvement in our patient.

As previously mentioned, HZO accounts for 10-20% of shingles cases in the United States. Ophthalmoplegia accounts for 7-31% of HZO cases. In a retrospective single-center analysis, Tsao et al. reported that each of the cranial nerve palsies causing ophthalmoplegia constituted 0.3% of the 330 HZO cases they reviewed [9]. Based on the statistics reported in the literature, our patient’s presentation constitutes a relatively rare HZO sequela. In the last decade alone, however, at least 10 case reports resembling ours were published, suggesting that HZO-related ophthalmoplegia may be more prevalent than reported. Further epidemiological studies are needed to establish the true prevalence of HZO-related ophthalmoplegia.

The etiology of extraocular muscle involvement in HZO is unclear. Evidence suggests that ophthalmoplegia may occur independently of the ganglion inflammation implicated in the pathogenesis of HZO [2]. It is possible that inflammatory processes in the surrounding tissues, such as perivasculitis and myositis, and/or muscular ischemia play a role in the development of the condition [6,10]. Other theories, explained by Shin et al., include direct viral cytopathic effect on the surrounding tissue, allergic response to the virus, or activation of another latent, neuropathic virus by HZO [11].

Despite the unclear pathophysiology, HZO-related ophthalmoplegia appears to be self-limited in most cases. Similar case reports have described the spontaneous resolution of symptoms within six to eight weeks [2,10,12]. Our patient fully recovered his extraocular function within eight weeks of developing HZO symptoms. Treatment with antivirals and/or steroids remains controversial given the self-limiting course of the ophthalmoplegia. Pharmacological treatment, however, may lead to prompt resolution of skin lesions, diminish viral shedding, and reduce the formation of lesions [13]. Our patient completed a two-week course of valacyclovir with the onset of HZO, about two weeks prior to developing ptosis and abducens nerve palsy. His only other prescribed medication was gabapentin for postherpetic neuralgia. His ptosis and abducens nerve palsy resolved without any specific, targeted treatment. Therefore, the progression of our patient’s symptoms is consistent with other previously reported cases.

## Conclusions

HZO manifests as a painful vesicular rash in the trigeminal nerve distribution that can progress to self-limiting ophthalmoplegia. The etiology of this sequela remains unclear. It most commonly results in oculomotor nerve palsy, whereas abducens nerve palsy, as seen in our patient, appears to be relatively rare. However, HZO-related ophthalmoplegia may be more prevalent than previously reported in the literature. This case report supports existing evidence of this condition’s favorable prognosis. The ophthalmoplegia may resolve spontaneously within weeks to months, without additional treatment beyond the initial antiviral therapy at the onset of HZO symptoms.

## References

[1] Liesegang TJ (2008). Herpes zoster ophthalmicus natural history, risk factors, clinical presentation, and morbidity. Ophthalmology.

[2] Shin MK, Choi CP, Lee MH (2007). A case of herpes zoster with abducens palsy. J Korean Med Sci.

[3] Shekhawat N, Talwar N, Stein JD (2019). Demographic and temporal variation in incidence of herpes zoster ophthalmicus in the United States population. Invest Ophthalmol Vis Sci.

[4] John AR, Canaday DH (2017). Herpes zoster in the older adult. Infect Dis Clin North Am.

[5] Minor M, Payne E (2022). Herpes zoster ophthalmicus. https://www.ncbi.nlm.nih.gov/books/NBK557779/.

[6] Niederer RL, Meyer JJ, Liu K, Danesh-Meyer HV (2021). Herpes zoster ophthalmicus clinical presentation and risk factors for loss of vision. Am J Ophthalmol.

[7] Nguyen V, Reddy V, Varacallo M (2022). Neuroanatomy, cranial nerve 6 (abducens). https://www.ncbi.nlm.nih.gov/books/NBK430711/.

[8] Kim SY, Motlagh M, Naqvi IA (2022). Neuroanatomy, cranial nerve 4 (trochlear). https://www.ncbi.nlm.nih.gov/books/NBK537244/.

[9] Tsau PW, Liao MF, Hsu JL (2020). Clinical presentations and outcome studies of cranial nerve involvement in herpes zoster infection: a retrospective single-center analysis. J Clin Med.

[10] Kreibig W (1959). [Zoster diseases of the eye]. Klin Monbl Augenheilkd Augenarztl Fortbild.

[11] Shin HM, Lew H, Yun YS (2005). A case of complete ophthalmoplegia in herpes zoster ophthalmicus. Korean J Ophthalmol.

[12] Bak CG, Jun DC, Kim JH, Kim HT, Kim SH, Kim MH (2002). A case of ophthalmoplegia caused by herpes zoster ophthalmicus. J Korean Neurol Assoc.

[13] Chaker N, Bouladi M, Chebil A, Jemmeli M, Mghaieth F, El Matri L (2014). Herpes zoster ophthalmicus associated with abducens palsy. J Neurosci Rural Pract.

